# Generation Victoria (GenV): protocol for a longitudinal birth cohort of Victorian children and their parents

**DOI:** 10.1186/s12889-024-21108-1

**Published:** 2025-01-03

**Authors:** Elizabeth K. Hughes, William Siero, Alisha Gülenç, Susan A. Clifford, Tony Frugier, Simon M. Hall, Jatender Mohal, Kathryn North, Natasha Zaritski, Sharon Goldfeld, Richard Saffery, Melissa Wake

**Affiliations:** 1 Murdoch Children’s Research Institute, 50 Flemington Road, Parkville, VIC 3052 Australia; 2 Department of Paediatrics, The University of Melbourne, Parkville, VIC Australia; 3 Melbourne School of Psychological Sciences, The University of Melbourne, Melbourne, VIC Australia; 4 Centre for Community Child Health, Royal Children’s Hospital, Parkville, VIC Australia

**Keywords:** Cohort Studies, Birth Cohort, Parents, Adulthood, Research Methodology, Data Linkage, Biological Specimen Bank, Population Health, Intervention studies, GenV (Generation Victoria)

## Abstract

**Background:**

In a world confronted with new and connected challenges, novel strategies are needed to help children and adults achieve their full potential, to predict, prevent and treat disease, and to achieve equity in services and outcomes. Australia’s Generation Victoria (GenV) cohorts are designed for multi-pronged discovery (what could improve outcomes?) and intervention research (what actually works, how much and for whom?). Here, we describe the key features of its protocol.

**Methods:**

GenV is a whole-population longitudinal platform open to all ≈150,000 children born within a two-year window and residing in the state of Victoria and their parents. GenV is guided by its 6 principles of Inclusivity, Collaboration, Sustainability, Enhancement, Systematised Processes and Value and enabled by large-scale biobanking, IT and novel high-throughput technology infrastructure. Successive recruitment phases were designed to maximise GenV’s inclusivity: (1) a smaller Advance Cohort from December 2020; (2) Newborn recruitment, with presence in 58 Victorian maternity services supporting face-to-face approach to parents of babies born 4 October 2021–3 October 2023; (3) Intensive remote recruitment to mid-2024 targeting those missed around birth; and (4) Ongoing indefinite enrolment of in-age children and their parents. Participants consent to universal (1) data linkage (e.g., state and federal physical and mental health, education, social, geospatial, ecological); (2) biosamples storage and use (e.g., residual clinical pregnancy and newborn screening, GenV-collected perinatal parent/infant saliva); (3) phenotypic and biosamples collection waves at child ages 6, 11 and 16 years, likely in schools and remotely for parents; and (4) opportunities for collaborative research integrated into GenV as a population registry (e.g., trials, natural experiments, depth subcohorts). Many participants supplement universal data with additional biosamples (e.g., infant stool, breast milk) and brief digital remote ‘GenV and Me’ assessments over the first 5 years. GenV will make all research data available, adhering to the principles of Open Science.

**Discussion:**

Launched in the COVID-19 pandemic and committed to diversity and inclusivity, GenV’s parallel consented child and pre-midlife cohorts will be positioned to help address today’s pressing issues such as chronic mental and physical health conditions, inequity, public health crises such as pandemics, and climate harm.

**Trial registration:**

ClinicalTrials.gov: NCT05394363; retrospectively registered 23 May 2022 (8 months into recruitment).

## Background

The mid-twenty-first century presents daunting challenges for researchers aiming to improve global wellbeing. Life expectancy appears to have peaked in diverse countries, with declines likely driven by the crises of chronic disease, global heating, pandemics, geopolitical instability and growing inequity [1–7]. We need innovative strategies to foster flourishing populations, prevent illness and address the complex, connected problems of our time, but the traditional ‘one question at a time’ research model fails to deliver solutions at the needed pace and breadth [8]. However, amid these challenges lies extraordinary opportunity. Technological advances in data, biosamples, imaging, and services enable research to operate faster, broader, deeper and at unprecedented scale. The vision of cell-to-society research [9] is now within reach.

**Fig. 1:**
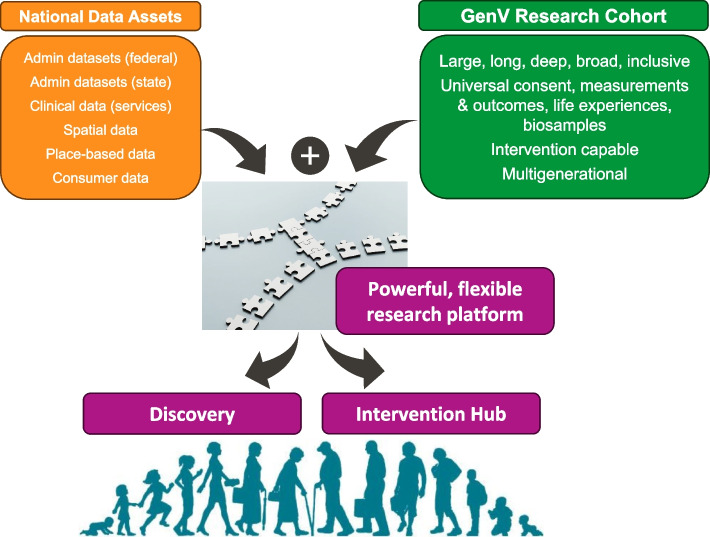
A research platform to speed up solutions for complex early and later life challenges

To help drive such solutions, we designed Generation Victoria (GenV), Australia’s largest-ever consented birth and parent cohorts, with the aim of creating a multi-use, problem-solving research resource to increase the speed, efficiency, scale, flexibility, and volume of research. In turn, this would help build a strong evidence base for better and more equitable prediction, prevention, detection and treatments (Fig. 1) that meets the needs of our people, services, and policymakers, and advances science.

The concept of GenV began in 2011 with a simple question: By working together, what single initiative would make the biggest difference to our children’s and our nation’s futures? The ‘Big Idea’ that emerged has since matured into GenV. The design of very large birth and parent cohorts was selected to efficiently focus both on early life (optimising growth trajectories, building resilience and minimising toxic stress for lifelong health) and pre-midlife (maintaining optimal health and functioning to avert or delay the chronic conditions that make up 85% of the burden of disease and contribute to 9 in 10 deaths [10, 11]). Health was conceptualised as more than the absence of illness, to encompass physical, mental and social wellbeing [12] of individuals and populations, capacity to adapt to challenges and leverage resources [13], and determined by multiple factors including social and environmental [14]. Equity and inclusion had to be at the heart of all decisions; we wanted GenV to represent a shift away from research that mostly benefits people least in need to a virtuous cycle of participation, applicability, and proportionate value return [15].

Inclusion of the full spectrum of diversity has informed many aspects of our protocol design. To our knowledge GenV is the only very large (> 20,000 children) longitudinal cohort internationally with recruitment not only in its country’s primary language, but also in multiple other languages. In this, and the decision to allow indefinite recruitment to those meeting our birth window criterion, we designed GenV to be welcoming to migrant and refugee families arriving beyond infancy who are typically never included in birth cohorts. Further, on reviewing phenotypic and performance measurement protocols of other very large recent cohorts, it appeared that none has yet been able to fully cater to the range of neurodiversity, disability, languages and cultures, disadvantage or domiciles for true inclusion. Finally, inclusion needed to go beyond GenV’s participants with a commitment to Open Science and support for underserved researchers and research domains.

As well as a resource for observational research, we have designed GenV to be capable of testing what strategies work, how much and for whom, including large-scale health, educational and social evaluations usually considered outside the reach of randomisation [16, 17]. This has profoundly shaped GenV, because an intervention-capable population platform requires (1) an explicit focus on Core Outcome Set measures needed to test efficacy and effectiveness, (2) participation not only across all population groups but across all services and all communities (hence GenV’s whole-of-state rather than national purposive sampling) and (3) rapid-fire, responsive waves to quickly provide definitive answers to big questions (hence our constraint of GenV’s birth window to two years). This would give academic, philanthropic, commercial and government funders and collaborators confidence that questions important to their priorities could achieve outcomes within their short cycles of 3–5 years.

By 2015, the global importance of having a cohort of the 2020s had risen with the cancellation of the US National Children’s and UK Life Studies, both of which had aimed to recruit 100,000 children [18, 19]. We were acutely aware of the problems they had encountered and the resulting international gap in mega-cohorts. This cemented our approach of a cohort time-limited in recruitment, embedded in trusted services, and light in touch for its participants. Yet at the same time, for GenV to be at the cutting edge of science and value, it still needed to include universal biosamples, all major exposure domains, services and outcomes (both phenotypic and experienced) relating to all major burden of disease, societal challenges, and wellbeing. These seemingly incompatible demands have required us to embrace universal-capable technologies for GenV-collected biosamples and data and to create the greatest value from the systems, data, and biosamples that already exist. These needs have driven GenV’s newborn sampling frame (the daily census of births created by the Victorian Infant Hearing Screening Program in every Victorian birthing hospital), its use of residual universal clinical biosamples, and our work to merge our consented cohort with the rich state and national data asset landscape (administrative, clinical, service, geospatial, others).

Our planning could not have foreseen the COVID-19 pandemic. To our knowledge, GenV is the only mega-cohort that proceeded to launch during this period. Although imposing a series of additional challenges, this uniquely places it not only to determine long-term population impacts of the pandemic itself, but also to address developmental origins of health and disease (DOHaD) via the extraordinary natural experiments that ensued in social and educational policy, health care, and environmental exposures [20]. This is compounded by mounting climate hazards of heat, fire, and flood disasters experienced at different times and places in our large state (population now approaching 7 million; geographical size comparable to the whole of the UK) since 2020 [21, 22].

**Fig. 2:**
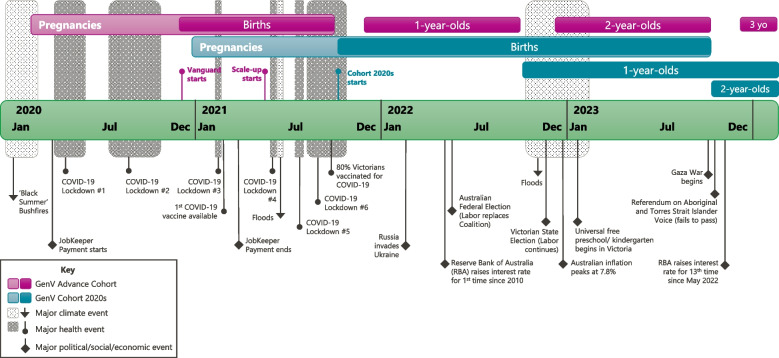
GenV pregnancies and births in relation to major climate, health and political/social/economic events

The GenV establishment Protocol presented here has been shaped by all the above influences (see Fig. 2). Table 1 summarises publications detailing aspects of GenV’s conceptual and methodological development; we refer readers to this published work for further detail.

**Table 1:** Key GenV peer-reviewed publications and Working Papers to date, providing additional conceptualisation and methodological detail

Topic Area	First author (year)	Citation	Description	Relevant Protocol Section
Conceptual	Davies (2020)	[23]	GenV’s research methodologies	Design
	Davenport (2020)	[24]	GenV’s focus areas	Data and Biosample Collection
	Hu (2021)	[20]	COVID-19 in pregnancy and birth cohorts	Introduction; Discussion
	Hughes (2020)	[25]	GenV’s parent consultations	Participant Involvement and Pilot Testing
	Nkyekyer (2021)	[26]	Maximising participant engagement	Participant-provided information
	Wake (2023)	[27]	GenV as a ‘solutions system’	Design
	Wang (2021)	[28]	GenV’s life course framework	Design
	Wyatt (2024)	[29]	Involvement of young people in health research	Participant Involvement and Pilot Testing
Measures	Clifford (2020)	[30]	GenV’s measures selection principles	Participant-provided information
	Musgrove (2022)	[31]	Core outcome sets for cohort studies	Data and Biosample Collection
Data & Biosamples	Hu (2022)	[32]	Accuracy of short-hospital record matching	Data Linkage
	Zhuang (2022)	[33]	Multi-omics analysis from newborn screening cards	Biosamples
	Camm (2024)	[34]	Collection of placental morphological data	Biosamples
Integrated Studies	Sung (2021)	[35]	Integrating registries in GenV	Integrated Studies
	Wake (2020, 2022)	[16, 17]	Embedding trials in GenV	Integrated Studies
	Wang (2020)	[36]	Scoping a special care nursery registry	Integrated Studies
	Wang (2023)	[37]	Study protocol for a special care nursery registry	Integrated Studies
Governance	Bell (2021)	[38]	Digital mega-studies as a new research paradigm	Design
	Davies (2020)	[39]	Large research-led partnerships	Design

For more detail on GenV’s peer-reviewed publications and for Working Papers see our website [40]

## Methods

### Design

GenV comprises very large longitudinal intervention-capable population-based cohorts of children and their parents. It is designed to encompass in the future:

- Consented cohort – Targeting all children living in the state of Victoria, Australia, and born in a 2-year period and their parents, with recruitment primarily soon after birth in GenV’s establishment phase but remaining open indefinitely for entry at any age;
- Biosamples – Curation and storage of residual universally collected biosamples, supplemented with collection of additional biosamples;
- Linked data – Ongoing access to extensive administrative, service, clinical, geospatial, and ecological datasets, including prospectively-collected prenatal and preconception datasets predating consent;
- GenV-collected data – Information collected from participants via GenV and/or in partnership with services; face-to-face direct assessment waves once at school conceptualised for child ages 6, 11 and 16 years (early, mid and senior school waves);
- Integrated studies – Collaborations that involve GenV participants in concurrent observational and interventional research that is embedded within or alongside GenV, subject to ethical approval and enacted agreements (e.g. data sharing, co-participation recruitment); and
- Open Science platform – The data and IT technology, systems and services needed to manage and make the GenV resource available to all future researchers for discovery and interventional research across all life stages.

**Fig. 3:**
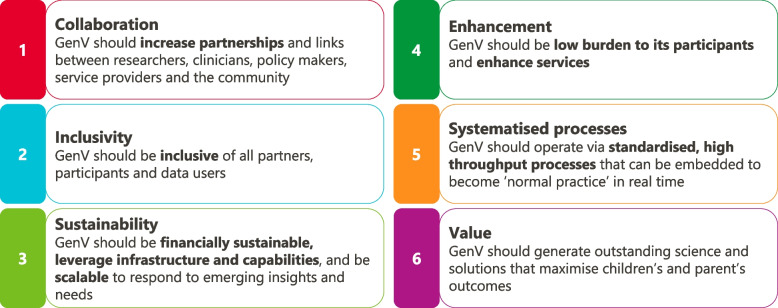
GenV’s guiding principles

**Fig. 4:**
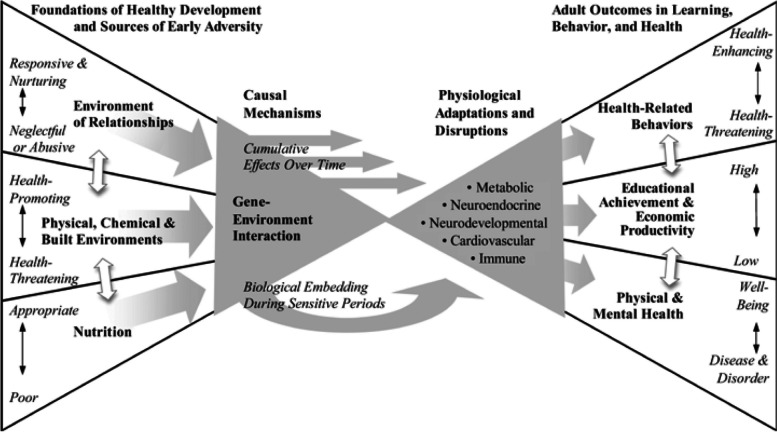
GenV’s primary life course framework. Adapted from Shonkoff J 2010 [41] with permission from John Wiley and Sons

**Fig. 5:**
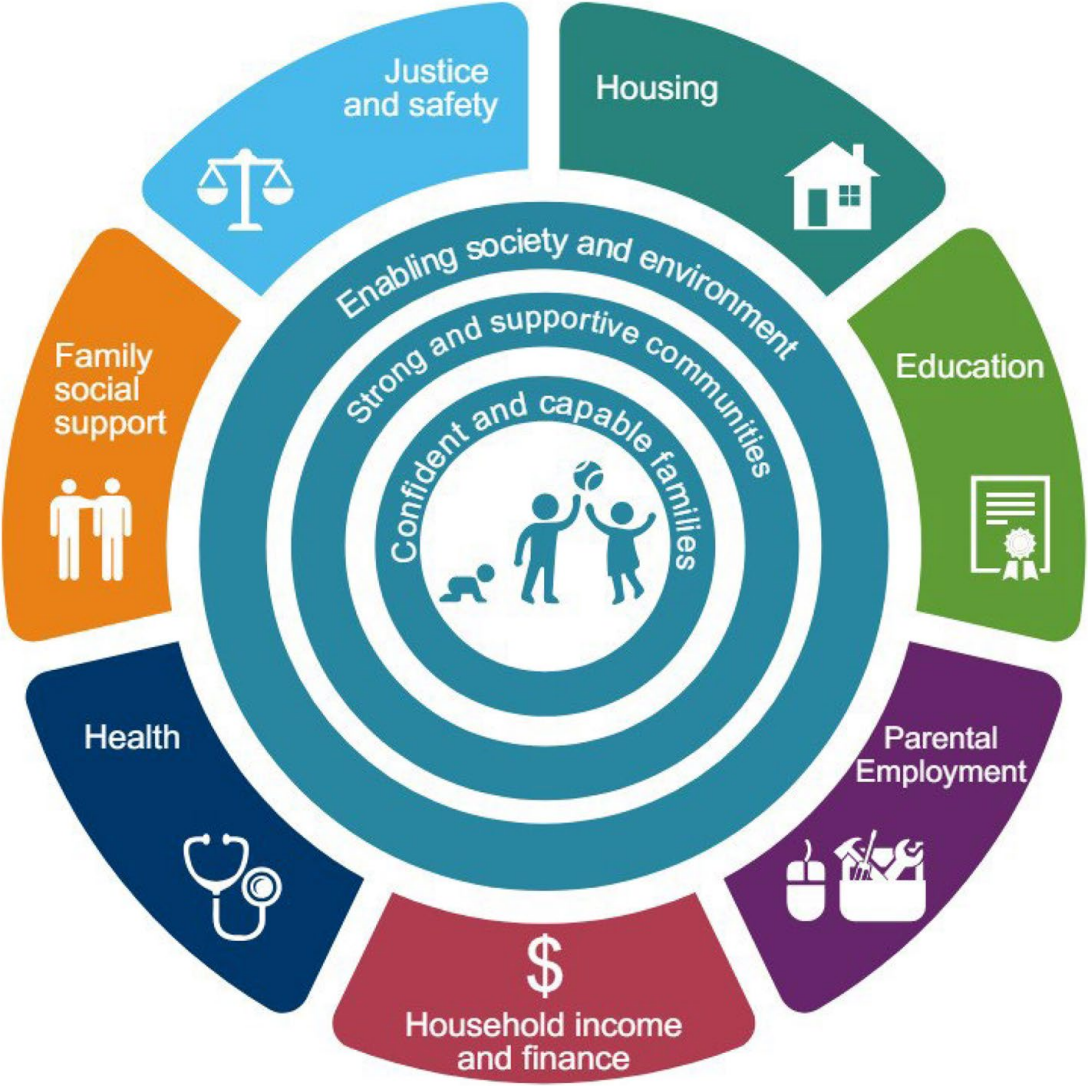
GenV’s secondary life course framework

All GenV activities are guided by its six principles (Fig. 3). Selection of exposures and outcomes are underpinned by GenV’s primary and secondary life course frameworks [28], based on frameworks adapted with permission from Shonkoff [41] and the Australian Institute of Health and Welfare [42], respectively (Figs. 4 and Fig. 5).

### Setting

GenV is based in Victoria, Australia. It is led from the Melbourne Children’s Campus by the Murdoch Children’s Research Institute (MCRI) with The Royal Children’s Hospital and The University of Melbourne, and in partnership with organisations across Victoria. Victoria has a population of over 6.6 million and annual births around 75,000 [43]. Of the total Victorian population, 29% are aged between 25 and 44 years old, 47% are in a married or de facto relationship, and families with children have an average 1.8 children [44]. The most common countries of birth (excluding Australia) are India, England and China; 30% of households speak a language other than English; and 1% of the Victorian population identify as Aboriginal or Torres Strait Islander [44].

### Participant eligibility

Children born from 4 October 2021 through to 3 October 2023 (approximately 150,000 births in Victoria), and their parents/guardians, are eligible. The index child must be living in Victoria at the time of their recruitment. The only exclusion criterion is absence of a person with capacity to provide informed consent or to consent in any of the multiple languages available (see Translations and Interpreting). Minimum participation is one child and one parent. All parents and guardians of the index child are eligible to join (e.g., mothers, fathers, biological, step, adopted, foster, surrogate, donor). All children of multiple births (e.g., twins) are eligible, as are siblings born within the eligible period. Children born in the eligible period outside Victoria and their parent(s) can join at any age if they move into Victoria, and those who leave Victoria can continue to participate, although both groups will be missing some information.

### Sample size

The sampling frame comprises an estimated ≈150,000 children and their ≈150,000 birth mothers, and ≈135,000 second parents (assuming that 90% of very young children have a second parent figure in their lives). Although we do not yet know the final uptake, current estimates suggest that by the end of the GenV establishment phase in 2024, we will have recruited 45,000–50,000 children plus their respective parents. A sample size of 50,000 children equates to projected sample sizes of approximately:

- N≈25,000: Second parents
- N≈50,000: Primary parents as individuals (by design the same as the number of children)
- N≈75,000: All adults as individuals
- N≈125,000: All GenV participants as individuals.

Table 2 gives an indication of the potential effect sizes that can be detected for a variety of health, wellbeing and behaviour conditions. Utilising reported prevalences available in 2018, estimates for the sample size calculations were based on an unmatched nested case–control design with four controls for each case. Minimum estimable odds ratios were calculated assuming 80% power to detect an association between a binary exposure variable and binary outcome, considering some of the less common conditions (i.e., 0.10 – 4.50% prevalence) and more common conditions (9.8% and 20.3% prevalence) shown in Table 2, at alpha = 0.05. The prevalence of the binary exposure variable in the control group was allowed to vary from 5–20%. All calculations were conducted based on the methodology of Dupont [45] using the epi.ccsize in R statistical software version 3.5.1 [46] specifying the use of an unmatched design. Although the intention is to have data for the full cohort in GenV, an unmatched case–control design was adopted as power is primarily driven by the number of cases. Therefore, these calculations provide an indication of possible odds ratios that may be estimated. For less common outcomes with 0.1% prevalence, such as bilateral moderate or greater congenital hearing loss, odds ratios of between 1.76 and 2.00 can be detected for exposure prevalence of 20% and 10%, respectively. Smaller odds ratios of 1.10 can be detected for exposures of 10% prevalence when the outcome prevalence increases to 4.5% (e.g., food allergy).

**Table 2:** Minimum estimable odds ratio detectable for different prevalence levels of exposure and example outcomes assuming four controls per case

Example	Outcome prevalence	Exposure prevalence	Minimum OR^a^ (150,000)	Minimum OR^a^ (100,000)	Minimum OR^a^ (50,000)	Minimum OR^a^ + (20,000)
Bilateral moderate or congenital hearing loss						
	0.1%	5.0%	-	-	-	-
		10.0%	2.00	2.28	-	-
		15.0%	1.85	2.08	2.69	-
		20.0%	1.76	1.98	2.54	-
Out of home care						
	0.8%	5.0%	1.44	1.55	1.80	2.34
		10.0%	1.32	1.40	1.58	1.97
		15.0%	1.27	1.33	1.48	1.82
		20.0%	1.24	1.30	1.44	1.74
Autism spectrum disorder						
	1.5%	5.0%	1.32	1.39	1.57	1.94
		10.0%	1.23	1.28	1.41	1.67
		15.0%	1.19	1.24	1.34	1.57
		20.0%	1.17	1.21	1.31	1.51
Otitis Media						
	3.7%	5.0%	1.20	1.24	1.35	1.57
		10.0%	1.14	1.18	1.25	1.41
		15.0%	1.12	1.15	1.21	1.35
		20.0%	1.11	1.13	1.19	1.31
Food allergy						
	4.5%	5.0%	1.18	1.22	1.32	1.51
		10.0%	1.13	1.16	1.23	1.37
		15.0%	1.11	1.13	1.19	1.31
		20.0%	1.10	1.12	1.17	1.28
Small for gestational age						
	9.8%	5.0%	1.12	1.15	1.21	1.34
		10.0%	1.09	1.11	1.15	1.25
		15.0%	1.07	1.09	1.13	1.21
		20.0%	1.07	1.08	1.12	1.19
Eczema						
	20.3%	5.0%	1.08	1.10	1.15	1.23
		10.0%	1.06	1.07	1.11	1.17
		15.0%	1.05	1.06	1.09	1.14
		20.0%	1.05	1.06	1.08	1.13

^a^Calculated using epi.ccsize in R based on [45].—indicates not estimable

### Recruitment

During GenV’s major newborn recruitment drive from October 2021 to October 2023, over 250 field staff undertook face-to-face recruitment up to 7 days a week at 58 maternity services where 99% of Victorian births occur. The Victorian Department of Health endorsed GenV via a letter from Victoria’s Chief Medical Officer to the Chief Executive Officers of all maternity services. GenV then worked closely with each maternity service to build support among staff, including their Aboriginal and Torres Strait Islander services where available. A large social awareness campaign aimed to raise awareness of GenV with expectant and new parents. This included a range of materials distributed via maternity and community services, social media, traditional media, and digital and outdoor advertising. Recruitment usually occurred on maternity wards in the days after birth, or shortly thereafter in special care nurseries, neonatal intensive care units, and outpatient infant hearing screening clinics. Each day notifications of births were received via the Victorian Infant Hearing Screening Program, the only centralised daily census of statewide births. A GenV recruiter visited families in their hospital room or clinic, guided the parent(s) through the information, obtained written consent, and collected intake data and samples. Recruiters used digital tablet devices to present the parent/guardian information statement and consent form (PICF) and capture a digital signature via REDCap software [47]. The PICF included a short video, drop-downs, and links to the GenV website. Paper forms were used if online systems failed, and for some language versions. If a second parent was not present at the visit, the recruiter requested their details to contact later.

As recruitment remains open indefinitely, parents who were missed or undecided at the hospital visit can join at any time by phone or self-guided online recruitment. During phone recruitment, a GenV recruiter explains GenV, answers questions, seeks verbal consent, and then sends an email or SMS with a personalised link to the PICF for signing. For self-guided recruitment, GenV sends a personalised link to the parent to invite them to join, review study information and complete their consent without any direct interaction with a GenV Recruiter. A toll-free phone number, email address, and webform link are available for those who have questions prior to or after consent.

#### Translation and interpreting

GenV aimed to implement a comprehensive translation and interpreting approach to give all eligible participants an equitable opportunity to join and actively participate, regardless of language spoken. Written recruitment materials and videos were translated into 25 common languages in Victoria. The selected languages cover an estimated 98.5% of the target population as indicated by parent use of interpreters for a statewide well-child service (informal communication). The translated PICF and intake survey were available in 5 languages other than English from the start of recruitment, with a further 20 rolled out between November 2021 and May 2023.

For face-to-face hospital and phone recruitment, GenV has used a variety of interpreting methods including bilingual recruiters, phone interpreting service (offering 190 languages), face-to-face interpreting services, digital interpreting using Google Translate via tablet, and bilingual family members.

While follow-up communications and surveys were initially available in English only, additional languages were implemented in 2023 and continue to be rolled out at time of publication.

### Scope of consent

Table 3 outlines the core consent components for GenV and the participant for whom the consent is required. All consent components are categorised into one of three levels:

1. Bundled general consent for several components – the minimum for participation in GenV
2. Additional item-by-item consent to specific components – optional for participation in GenV
3. Separate consent by the second parent/guardian.

**Table 3:** GenV consent components

**Consent component**		From
**General consent**—**for primary parent/guardian (usually mother**^**a**^**) and child**		
1	Access to/retrieval of health, education, social and place-based information including: a. Administrative data spanning physical and mental health, education, social, births and deaths, geographic b. Service & clinical data including individual (e.g. hospitals, GPs) and collated, Electronic Health Records (EHRs), digital data (e.g. MRI scans)	P1
2	Ongoing contact by GenV to a. Collect new information b. Provide information to participants c. Collect additional consent (eg participation in collaborating studies)	P1
3	Updating participant contact details from parent or via public records or information held by agencies	P1
4	Unspecified use of data for future ethically-approved research	P1
5	Sharing and 2-way exchange of data with approved GenV users (e.g. Integrated Studies)	P1
6	Retrieval and use of residual clinical biosamples stored pre/postnatally	P1
7	Collection and use of new biosamples (infant stool swab, breast milk)^b^	P1
**Item-by-item consent**—**for primary parent/guardian (usually mother) and child**		
8	Genetic research	P1
9	Collection and use of new biosamples (saliva)	P1
**Separate consent**—**for 2nd parent/guardian (usually father)**		
10	Participation covering all of the above for 2nd parent	P2
11	Collection and use of new biosamples from 2nd parent (saliva)	P2

P1 = parent consenting on behalf of themselves and the child (usually the mother). P2 = parent consenting for themselves only (usually the father). ^a^ Only the mother who carried the child can consent for access to her records and retrieval and use of her pre/postnatal biosamples, except in the rare situation where the mother herself has a guardian. ^b^ Introduced October 2022

Length of participation is open-ended and will continue (including after death) until the participant withdraws or the study closes. Participants are free to withdraw at any time and can choose whether already-collected data and samples continue to be used for research. Children will assume responsibility for their continued participation at an age of decisional capacity, likely to be 14–18 years.

### Data and biosample collection

**Fig. 6:**
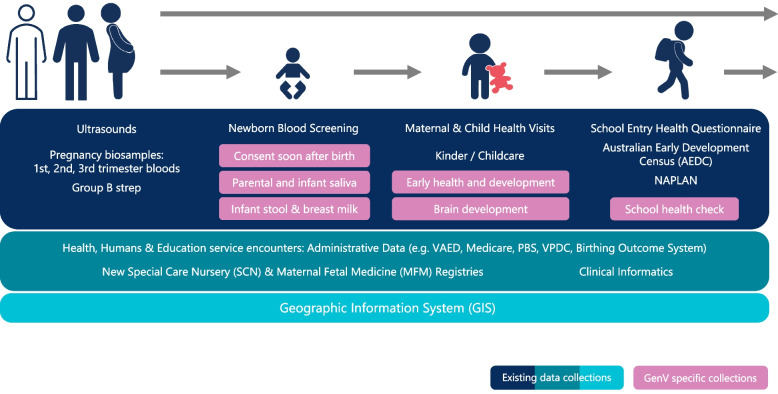
GenV data and sample collection journey. *Notes.* PBS = Pharmaceutical Benefits Scheme; NAPLAN = National Assessment Program – Literacy and Numeracy; VAED = Victorian Admitted Episodes Dataset; VPDC = Victorian Perinatal Data Collection

Figure 6 depicts the GenV data and biosamples collection journey. All data are collected or accessed digitally, with paper backups where necessary and possible. Data may include images, videos, traces and digital objects that GenV either collects directly (recruitment survey, *GenV and Me* digital assessments, school-based waves) or accesses via routine care (e.g., pdfs of written or typed documents, magnetic resonance imaging, ultrasound, echocardiogram). It will also include location information (e.g., participant residence, schools, geographical areas [48]), along with environmental attributes across both geographic space and time. Notably, whole-cohort follow-up data will be achieved by data linkage and major 5–6 yearly face-to-face assessment waves, while participant-provided data between these waves is anticipated to be completed only by a subset of participants.

### Participant-provided information

Parents complete optional brief digital ‘*GenV and Me’* assessments to provide high-value data not available elsewhere (see Table 4). The custom-designed digital platform was co-developed with a digital health company (WeGuide.health). Participants can choose to use either the *GenV and Me* App or website on any smartphone, tablet or computer. To support universal participation, it works on widely used operating systems (including OS versions dating back at least 3 years), meets disability accessibility standards (WCAG v2.1 Level AA, e.g. compatibility with braille keyboards and text-to-speech screen readers) and allows passwordless login. At time of writing, it is available in 5 left-to-right languages (English, Simplified Chinese, Vietnamese, Burmese, Punjabi), with two right-to-left languages (Arabic, Dari/Farsi) soon to be added.

**Table 4:** Recruitment and ‘GenV and Me’ assessment constructs

	**R**	**3 m**	**6 m**	**9 m**	**12 m**	**18 m**	**2 yr**	**2.5 yr**	**3 yr**	**3.5 yr**
**Demographics**	C,P	C,P			C	P		P		
**Tier 1: overarching generic constructs**										
Pregnancy, birth & post-pregnancy	C,P	P		C		P				
Global health	P	C,P	C,P	C,P	C,P	C,P	C,P	P	C,P	P
Health conditions, illnesses, injuries, pain		C,P	C,P	C,P	C,P	C,P	C,P	C	C,P	C
Health Related Quality of Life				P			C,P		C,P	
Disability and SHCNs		C,P	C,P	C,P		C,P	C,P	C,P		C,P
Development^a^		C	C,P	C	C,P	C,P	C,P	C	C,P	C
**Tier 2: core phenotypic constructs**										
Respiratory									C	
Dysmorphism					C		C			C
Body Size and composition	P	C	P		C,P	C	C,P		C,P	
Skin & Eyes								C,P		
Oral Health									C	
Motor (Fine and gross)		C	C						C	
Language, Speech and Communication	P^b^				C,P		C,P		C,P	
Mental health	P	P	P	P	P	P	P	P		P
Temperament, behaviour, happiness		C		C	P	C,P	C,P	C,P	P	C,P
Life satisfaction			P		P		P			P
Self-regulation		P		P		P				
Sleep, fatigue, energy	P	C,P		C,P			C,P			C,P
Social		P					C,P			P
Stress and Coping									P	
**Tier 3 + : other constructs**										
Feeding, nutrition and diet	P	C,P	C	C	C	C	C		C	C
Allergy and Eczema					C	C	C		C	
House and household, family life and wellbeing	P	P	P	P	P	P	P	P	P	P
Parenting		P		P		P	P		P	
Relationship with partner				P			P		P	
Employment							P		P	
Society							P			
Health priorities			P	P	P	P	P			

C = parents’ report about the child; P = parents’ report about themselves; R = Recruitment survey; SHCN = Special Health Care Needs

^a^P refers to parents’ report of their concern about the child’s development, not their own

^b^Parents’ spoken languages

^c^Birth parents’ sleep position during pregnancy

*GenV and Me* is designed to remotely capture survey items, diagnoses, life events, photos, videos, adaptive tests and direct assessments, collectively spanning cognition, physical and mental health, development, functioning, growth and dysmorphology. *GenV and Me* thus allows low-burden remote assessment of the ‘ePhenome’ from the large, socioeconomically-diverse and geographically-dispersed GenV cohort. Following a survey at recruitment, all parent participants are sent *GenV and Me* invitations via email, SMS and push notification four times a year in the child’s first year of life, then biannually through the preschool years. All parents receive separate modules about themselves and about their child to complete; the GenV self-identified ‘primary’ parent provides additional child information to the child’s other parent/s. At a later date, we expect child-completed modules to be added.

We have commenced planning for the fourth major universal component of GenV, which is the major face-to-face outcomes waves that will support a range of child and parent direct assessments, monitoring and wearables at approximately 6, 11, and 16 years of child age. The content and funding for these school-age collections are yet to be confirmed and are thus not covered here. GenV may also work with services in the future to collect data from participants.

#### Data linkage

To optimally advance prediction, prevention and treatments, very large administrative datasets must be merged with data from well-phenotyped cohorts. GenV obtains broad consent to access past, current, and future clinical and service records (e.g., hospitals, pathology services, general practitioners) and administrative datasets (e.g., health, social, education, neighbourhoods). At the time of writing, we are planning for future integration of the GenV asset with two whole-population secure data assets that combine data for our state (via the Centre for Victorian Data Linkage) and for our nation (Person Level Integrated Data Asset, held by the Australian Bureau of Statistics). Collectively, these assets span perinatal morbidity, births and deaths, ambulance, primary care, emergency and hospital utilisation, mental health, public health, community health, elective surgery, human services, education, government payments, income and taxation, employment and population demographics.

#### Biosamples

**Fig. 7:**
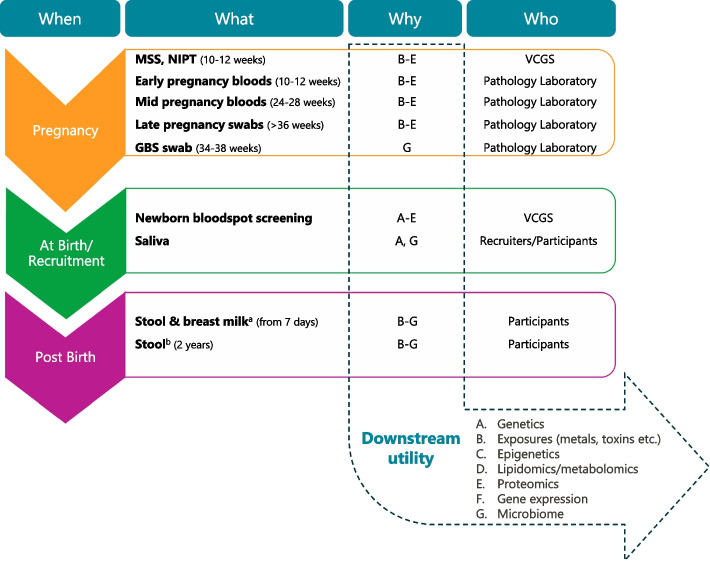
GenV biosamples and their downstream Utility (A-G). *Notes.* GBS = Group B Streptococcus; MSS = Maternal Serum Screening; NIPT = Non-invasive Prenatal Testing; VCGS = Victorian Clinical Genetics Services. ^a^ Implemented October 2022. ^b^ To be implemented late 2024

Figure 7 summarises the biosamples available to GenV and their downstream utility. These fall into two categories, i) residual clinical biosamples (longitudinal pregnancy serum or plasma, group B Streptococcus (GBS) vaginoanal swabs, and newborn screening blood spots); and ii) participant-provided biosamples (child and parent saliva, infant and 2-year-old stool, and breast milk).

The Victorian Clinical Genetics Service (pregnancy first and second trimester fetal screening and newborn blood screening samples) and eight Victorian pathology providers (pregnancy serum and/or plasma and third trimester GBS swabs) store excess residual clinical samples in 2D-barcoded tubes, identifiable only to the custodial pathology provider, at -80 °C at the Melbourne Children’s Bioresource Centre (see below). These samples remain under custodianship of each pathology provider until participant consent for GenV is demonstrated and custodianship is transferred to GenV. Parents also consent for GenV to access clinical assay results from pathology providers for these and future biosamples.

GenV also collects samples directly from participants. When parents provide additional specific check-box consent to saliva collection (over and above the broad bundled consent), GenV collects saliva samples from the parent/s and child at the recruitment visit in hospital, or by post if recruited by phone or self-guided recruitment. Saliva is collected using DNA Genotek ORAcollect™ swabs and stored at -80 °C. During the final 12 months of hospital-based newborn recruitment, parents were also offered, under the bundled consent, a pack containing two kits for at-home collection of an infant stool swab (commercial kit from Microba©) and up to 5 mL breast milk sample (5 mL tube containing a custom-made freeze-dried preservative developed and prepared by University of Western Australia in collaboration with GenV). Parents were asked to collect both samples at child age 7 days if possible, or at the earliest possible point thereafter but with no hard upper age limit. Parents posted both kits back to GenV in a prepaid envelope at ambient temperature. Upon receiving, GenV’s lab team processed and stored the samples at -80 °C. Published Microba data attest to stool swab stability at temperatures of up to 50 °C for 4 weeks [49]. Breast milk samples have been tested for stability in the laboratory at different temperatures over 35 days up to 37 °C. Quality control analyses of GenV samples by the University of Western Australia have shown 18 different milk components within published reference ranges. Data generation is ongoing with a publication in development.

A follow-up stool sample collection is planned for around age 2 years from late 2024. This will be offered only for participants who previously returned an infant stool sample.

### Integrated studies and trials

GenV can be considered not only as a cohort study but also as a ‘basket’ registry spanning many issues, groups and clinical conditions at universal or targeted population level. Whereas classic clinical registries consider only one condition [35], GenV can meaningfully consider issues of low incidence/prevalence and co- and multi-morbidities. This allows us to travel from simply observing how children mature and pre-midlife adults age to optimising a range of individual and “stacked” interventions to shape growth, development, ageing and thence our population’s life course [16, 17, 50].

GenV supports collaborative co-participation in other ethically approved research projects, including trials, registries and cohort studies. Co-participation means that a participant is not primarily in GenV or primarily in another study; rather, participants have expressed their interest in being part of both studies. Invitations to collaborating studies may be facilitated by GenV during recruitment or at a later contact, and vice versa. For example, GenV or the collaborative study inform eligible participants of the other study, or obtain consent to refer participants directly.

Participants who join both GenV and a collaborating study can consent to data sharing between the two studies. This increases the value of both studies and may reduce research costs, effort, and duplication (i.e., where collaborating studies can access rich GenV-collected and linked data, and/or GenV-collected biosamples and bioassay data). With full attention to privacy and governance, these assets allow the study of pre-existing and long-term confounders and effect modifiers, as well as longer or broader outcomes than typically possible by a single study. Additionally, they support impact modelling of outcomes to the Victorian population.

Beyond these parallel collaborations, GenV is designed to support very large, embedded trials and health services research such as new population screening workflows and health economic evaluation [16, 17, 51, 52]. Yet to be developed, the GenV Intervention Hub will aim to address critical questions not otherwise feasible and with high likelihood of sizable impact and implementability, as conceptualised in our prior publications**.** Its population registry infrastructure is intended to increase the size, speed, number and impact of early and pre-midlife clinical trials delivered at scale and drawing on > 125,000 Australian children and adults.

More information about collaborating with GenV and GenV’s current Integrated Study collaborations can be found in previous publications [16, 17] and on our website [40].

### Data storage and access

#### Core datasets

Core datasets within the scope of GenV comprise all GenV-collected and extracted data, biosample assay results, geographic information, and linked datasets (e.g., clinical, health, education, social and services record data). Integrated studies can contribute their own data to GenV, and research conducted using GenV data will feed back into the data resource to continually enhance the available data. GenV research data may interface with or enter alternate systems with comparable security and privacy controls (e.g., into secure online space to access linked datasets unable to be released by custodians).

#### Accessing core datasets

GenV is committed to ensuring data are made readily available to all bone-fide researchers and analysts (e.g. policy, services) under FAIR [53] and Open Science [54] principles, while also needing some cost recovery to support the sustainability of GenV. Data access will be assessed based on the ‘Five Safes’ framework: safe people, projects, settings, data, outputs [55]. Data from any given collection point will usually be made available only when collection for that point has ended (e.g., for the *GenV and Me* 12-month assessment, when all children have passed the age of 12 months and their parents had the opportunity to respond). Exceptions for early data release may include preliminary analyses internal to GenV, responding to public health, climate, or other emergencies, and necessary data for some integrated studies.

Data catalogues and enriched metadata will provide context and explanations of the data assets available to all users. Access processes and approval criteria are currently being developed to provide tiered access levels, allowing the use of more sensitive data (with special licence) under greater restrictions. GenV will use a combination of systems, policies, and processes to implement a data and biosample access framework. Under the same rules, users from commercial companies can access the data to conduct research with public benefits. Licenced users will access de-identified data, with confidentialising techniques applied as needed.

In the future, data may be accessible through various channels, depending on data sensitivity, the requesting entity, and research design. These options include virtual access within a confined and secure environment, electronic download of predefined or individually curated datasets, secured and approved point-to-point integration, access via approved third-party linkage services, national data sharing services, physical media, and other approved methods.

#### Biosamples storage and access

Biosamples are stored at the Melbourne Children’s Bioresource Centre. This state-of-the-art, high throughput, fully automated biobanking facility receives, curates, processes, stores, retrieves and distributes biosamples for bioassays. Our biobanking facility, purchased and commissioned by GenV, includes two automated liquid handlers (Microlab STAR and easyBlood STARlet) and the Hamilton® BiOS M10 -80 °C robotic storage system, the latter open for storage of samples from other projects. GenV uses OpenSpecimen (Krishagni Solutions Pvt Ltd) as its biosamples management system.

Bioassays may be conducted by GenV or by collaborators. GenV is developing bioassay policies, processes and user protocols, which will cover priorities, funding, depletability, and breadth and benefit of resulting research for future human health. Data and leftover samples must return to GenV in an agreed timeframe to benefit all researchers. To maximise efficiency and minimise batch effects, an assay will usually be undertaken for the entire GenV parent and child cohorts, and only once all samples from a given collection point are available. Exceptions might involve assays for late-enrolling individuals, such as school-aged children, who could provide baseline saliva samples and access to their stored newborn blood spots. These cases may require catch-up batches or result in missing data for priority assays. Assays for participant subsets may be considered from additional biosamples collected by integrated studies but not from GenV-collected biosamples. Bioassay data will enter GenV datasets like all other data (as above).

#### Data security and protection

The security and protection of data held by GenV are paramount and integral to its design and operations. Data are safeguarded through the adoption of key architectural principles in the design of storage and access protocols. Principles applied to enhance data security include:

- Minimising data movements across system boundaries
- Storing identifying information only where needed
- Separating identifying information from research data whenever possible
- Minimising data replication
- Encrypting data both in motion and at rest
- Adopting obfuscation principles.

Additionally, to ensure the ongoing security of data within GenV, we follow the Australian Cyber Security Centre’s recommended strategies for mitigating cyber security incidents [56].

#### Return of data to participants

At recruitment, participants are informed that they may request to access their personal information and correct any errors or omissions. Participants are also informed that GenV will not routinely return individual results; among other reasons, this approach reflects the brevity of the recruitment contact, GenV’s likely long timeframe, and the non-clinical nature of GenV measures. However, in the future GenV may give short on-the-spot reports of meaningful results and serious obvious incidental findings to participants (± provider) following direct or digital assessments. GenV may also ‘show back’ answers from earlier surveys to allow participants to confirm whether health or other events are ongoing or resolved. GenV communicates regular updates about GenV to participants via its newsletters and website to highlight studies and their findings. In the future, GenV aims to enable participants to query and visualise group data online (without compromising privacy).

### Research ethics and governance

GenV was granted ethics approval by The Royal Children’s Hospital Melbourne Human Research Ethics Committee (HREC #2019.011). Research governance approval was granted by each birthing hospital site’s Research Governance Office or appointee (e.g., Chief Executive Officer). Parents provide written informed consent for their own and their child’s participation in GenV. The HREC granted waivers of consent for transfer of limited data from the Victorian Infant Hearing Screening Program to GenV to identify and locate births and to contact families missed in hospital, and for pathology laboratory storage of antenatal biosamples prior to transfer of custodianship to GenV. GenV undertakes regular privacy impact assessments to ensure compliance with relevant legislation and to identify and mitigate any risks related to personal information. GenV’s Ethics and Governance Working Group comprises researchers, ethicists, and lawyers, and advises on matters related to the ethical and legal conduct of GenV.

### Governance structure

GenV’s governance structure ensures its objectives are delivered with quality, ethical standards, regulatory compliance, accountability, transparency in decision making, timeliness and within budget. Governance is reviewed periodically and adjusted to meet GenV’s needs. As of 2024, GenV is led by two co-Directors (Science and Cohort; Platform and Operations), supported by two Deputy Directors and a Program Manager. GenV’s streams (Cohort; Solutions Hub; BioDiscovery; Data; Platform and Operations) each have responsibility for design and operations. GenV is ultimately governed by the MCRI via the GenV Steering Committee, which ensures that the development, deployment and operations of GenV are successfully implemented and that GenV's benefits are realised for MCRI, stakeholders, and the community. Reporting to the Steering Committee, GenV’s Co-Directors and Leadership Team are responsible for broad decision-making about the conduct of GenV, while day-to-day management and decision-making is the responsibility of the GenV Management Group. Because of its size and scale, GenV also has a formal framework of risk, finance, and change controls, each with its own committee and MCRI oversight. Scientific direction is shared between GenV’s Cohort, Solutions Hub, BioDiscovery, and Data streams, with extensive input from advisory committees such as the Investigator and Strategic Partnerships Committees, and Working Groups.

### Participant involvement and pilot testing

Several pilot studies and consultations were conducted to inform the design of GenV. In 2014, we held three focus groups with parents to seek feedback on concerns and expectations in relation to a proposed large population longitudinal birth cohort study. This was followed in 2015 with a pilot study of recruitment of parents shortly after birth at a large metropolitan maternity hospital. Further parent consultations have included: an online survey of 100 parents in 2018 to seek views on access to children’s data for research; focus groups in 2019 to explore understanding, attitudes, and decision-making behaviours relevant to GenV with attention to differences between regional and metropolitan parents; repeat focus groups in 2020 following the COVID-19 pandemic onset with broad representation of location, employment, education, disability, and ethnicity; and a survey of 500 expectant parents and parents of young children in 2019 to assess the clarity, usefulness, comprehension and impact of the GenV recruitment information [25]. From 2023, all parents participating in GenV are invited to join the GenV Participant Advisory Panel to provide feedback on their experience of GenV and give input into proposed future activities.

Prior to launching statewide, GenV commenced ‘Vanguard’ recruitment from December 2020 at the Joan Kirner Women’s and Children’s Hospital, a large public metropolitan hospital serving a diverse population in Melbourne’s west. This provided an opportunity to refine recruitment procedures and evaluate the acceptability and recruitment impacts of several alternative consent components (e.g., inclusion vs exclusion of saliva and stool samples). Over a 5-month period from May 2021, GenV scaled up recruitment to all other sites across the state in a stepwise manner. Recruitment of children born from 5 December 2020 to 3 October 2021, and their parents, closed in July 2022. The participants of the’Vanguard’ and ‘Scale-Up’ cohorts jointly form GenV’s ‘Advance Cohort’. They are ongoing participants of GenV, although with less complete data.

### Community and expert engagement

GenV formed several working groups and consulted a diverse array of professionals to advise on its design and implementation. These included experts in pregnancy and newborns, biosamples, genetics, -omics, geospatial research, health services, registries and trials, epidemiology and biostatistics, universal service design and operations, and social marketing and communications. GenV has also benefited from ongoing and extensive input from groups representing Aboriginal and Torres Strait Islander families, those from culturally and linguistically diverse backgrounds, LGBTQIA + communities, and numerous peak bodies. Further information on GenV’s working groups can be found on our website [40].

## Discussion

Here we have presented the key features of GenV’s protocol as it stands towards the end of its establishment (newborn recruitment) phase, with additional detail available in our registration and published peer-reviewed and working papers. With an inclusive ‘cell to society’ philosophy [9] and shaped by global circumstance, GenV is creating a system of powerful research platforms designed to speed up and scale solutions for complex early life and later life challenges with equity and inclusivity as its heart (see Fig. 1).

Every major cohort has both strengths and limitations that reflect trade-offs in choices. Approaching a sample size of around ~ 50,000 children and ~ 75,000 parents recruited from birthing hospitals across an entire Australian state and supported by families, national peak bodies, policy and services, GenV has already delivered a perinatal cohort capable of modelling impacts and solutions for contemporaneous issues of global burden and multimorbidity. Its design encompasses early and pre-midlife diversity, consent, universal ante/perinatal biosamples, extensive data linkage, and experiential and phenotypic data. Ongoing recruitment targeted to priority populations and emerging issues will further enrich case-cohort and health, social, and educational economic value. Confirming its fitness for purpose, GenV has already leveraged > $30 million in competitive research projects involving over 150 collaborators even before establishment recruitment is complete. Most relate to new population-scale screening, diagnostic and statewide registry paradigms, with applications in train for GenV as a ‘registry’ for trials also at population scale.

While GenV has succeeded in many of its aims, it has also faced significant challenges that may have impacted uptake. The onset of the COVID-19 pandemic brought strict hospital policies and procedures including earlier discharge; shortened, distanced and/or contactless visits; wearing of full personal protective equipment including face shields; prohibition of staff working across multiple sites; and, at some sites, a complete lock out of GenV field staff. Widespread flooding in late 2022 [21] blocked roads needed for staff to access sites, and highly-publicised data breaches of large Australian telecommunications and health insurance companies [57] increased public concerns regarding data security with temporary downturns in consent rates. Nonetheless, GenV’s uptake to date suggests it will ultimately equal, if not exceed, that of other mega-cohorts including the Danish National Birth Cohort [58], the Norwegian Mother and Child Cohort Study [59], the Japan Environment and Children’s Study [60], Born in Guangzhou [61] and the Korean Children's Environmental Health Study [62] (as a percentage of all births in the region/country over the recruitment period).

We will soon have offered GenV recruitment to every Victorian baby born in our 2-year window. Beyond this, recruitment remains open to in-age children and their parents/guardians indefinitely. GenV’s ‘Door’s Always Open’ strategy will ensure the continued growth of the cohort via, for example, contacts with childhood services, referrals from integrated studies, and potentially a major intake at our early school face-to-face wave. We will publish a cohort profile in due course, noting that preliminary analyses already indicate that GenV’s demographic profile closely resembles that of all births in the state of Victoria for the two years of statewide recruitment.

When cohorts start early enough, run for long enough, are large enough, and reflect all populations to whom the findings will be applied, they can support world-class health and medical research, shed light on mental, physical, cognitive and social issues and support targeted solutions for the complex issues children and adults face today. Of our four core elements, GenV’s consented cohort and universal ante/perinatal biosamples are already a reality; extensive data linkage is in progress; and the early school phenomic wave (representing outcomes of the early years and transition to middle childhood [63]) is in planning for 2028–29. GenV’s first data and biosamples release is expected to open to research applications in late 2025, while expressions of interest to collaborate are open, encouraged now and can be made via our website [40]. With the GenV resource maturing, it will soon be ready to support new discoveries, understandings and solutions that benefit children and their parents.

## Data Availability

No datasets were generated or analysed during the current study.

## Abbreviations

GenV: Generation Victoria
GBS: Group B Streptococcus
HREC: Human Research Ethics Committee
MCRI: Murdoch Children’s Research Institute
MSS: Maternal Serum Screening
NIPT: Non-invasive prenatal testing
PICF: Parent/Guardian Information Statement and Consent Form
